# Wear Induced Failure of Automotive Disc Brakes—A Case Study

**DOI:** 10.3390/ma12244214

**Published:** 2019-12-15

**Authors:** Ali Mohammadnejad, Abbas Bahrami, Majid Goli, Hossein Dehbashi Nia, Peyman Taheri

**Affiliations:** 1 Department of Materials Engineering, Isfahan University of Technology, Isfahan 84156-83111, Iran; aliaria1997@yahoo.com (A.M.); a.n.bahrami@cc.iut.ac.ir (A.B.); majidgoli@yahoo.com (M.G.); h.dehbashi@pa.iut.ac.ir (H.D.N.); 2 Department of Materials Science and Engineering, Delft University of Technology, Mekelweg 2, 2628 CD Delft, The Netherlands

**Keywords:** failure, disc brake, automobile, wear

## Abstract

This paper investigated a failure in a ventilated disc brake in an automobile. The failed brake disc had been in service for approximately 10 years. The observed failure was in the form of radial cracks that appeared to have initiated at the outer edge of the disc brake. The cracks were rather straight with no branching. Optical microscope, scanning electron microscope (SEM), and energy dispersive X-ray spectroscopy (EDS) were used to study the microstructure of the failed disc. Vickers microhardness test was also used to evaluate the hardness of the samples. Results showed that the root cause of crack formation, in this case, was related to the excessive wear in the brake disc. Different wear mechanisms, namely abrasive and adhesive wear, were recognized in the failed specimen. Moreover, the worn surface in some areas was covered with fine oxide particles. These particles appeared to have a significant contribution toward abrasion. To further understand the wear mechanisms, pin-on-disc experiments were also conducted on the samples. Results of the pin-on-disc experiments were compared and correlated to the results obtained from the failed brake disc.

## 1. Introduction

Automotive disc brakes are known to experience severe working conditions under thermal fatigue, wear, and heavy mechanical loadings. Amongst different materials, cast iron is the most widely used material in disc brakes, since it has a unique combination of excellent wear resistance and a superior heat transfer coefficient [1]. Different parameters including alloying elements and graphite morphology, affect tribological behaviors of cast iron. There are several studies on the effects of graphite morphologies and alloying elements on the wear resistance of gray cast irons [2,3,4,5]. Silicon, chromium, and manganese are known to have a positive attribution to the wear resistance of cast irons. It is postulated that higher hardness and tensile strength in combination with less wear loss can be achieved by adding silicon and chromium to gray cast iron [6,7,8]. Further to the chemistry and microstructure of the alloy, abrasion and wear test variables also have prominent attributions to the wear behavior of gray cast irons. Whether the test is carried out in dry or lubricated conditions, the velocity of counter-moving parts during the test and applied load all have controlling effects on the wear behavior of alloys. For instance, gray cast iron shows much better wear resistance in lubricated conditions, obviously because of the lower friction coefficient [9,10,11,12]. Gray cast iron alloys are known to have a self-lubricating characteristic due to the presence of graphite flakes in their microstructures, making them an appropriate choice for sliding components in mechanical assemblies and installations [3,13,14]. That is why cast irons are still by far the most widely used material in disc brakes in trains and automobiles. Given that the disc brake is an essential component when the safety of passengers in the vehicle is a concern, understanding the degradation mechanisms of this component is a very key issue in producing more reliable and safer vehicles [15,16]. The degradation and failure of disc brakes can be very complicated because of the simultaneous contributions of mechanical loading, surface conditions, and thermal stresses. The fact that disc brakes experience dynamic loading conditions makes the interpretation of failures even more complicated. It is postulated that the surface spallation, formed during abrasion, creates spots on which cracks can be initiated. Further loading causes crack growth and failure of the disc [16,17,18]. Gao et al. [19] reported that the reason for crack formation and growth is the fatigue due to thermomechanical cyclic loading. This paper investigated a failure in a ventilated disc brake in an automobile and we presented a systematic and comprehensive assessment of the failure, considering the microstructure and loading conditions.

## 2. Materials and Methods

Samples for microstructural and mechanical properties characterizations were cut from a failed disc brake, using electro-discharge machining (EDM). Optical microscopes, scanning electron microscope (SEM, Philips, Eindhoven, The Netherlands), and energy dispersive X-ray spectroscopy (EDS, Cu Kα) were used to study the microstructure and wear mechanisms in the failed disc. The fracture surface of the failed specimens was also studied using SEM. The hardness of specimens was measured using a Vickers micro-hardness test (force 100 g with loading time 15 sec, according to ASTM E384-17). A laser profilometer was used to investigate the surface topology of failed samples. To further investigate the correlation between the microstructure and wear behavior of samples, a pin-on-disc wear test was conducted on samples of 50 mm diameter at ambient temperature, using a hardened AISI 52100 steel (Fe-1.0C, 0.4Mn, 0.15Si, 0.02S, 0.01P, 1.5Cr, in wt%) pin, with 5 mm diameter and hardness 64 RC. The specimens were taken from the disc. Wear tests were performed on the side which was not in contact with the pad. First, some trial wear tests with different loads were conducted to find the load at which the wear test yielded results in a reasonable time. Loads were changed in the trial tests from 10 to 60 N. Each test was run for 50 m. In the end, tests were carried out with 20 and 40 N loads. Results showed that experiments with loadings below 10 N took a very long time to see minor changes in the specimen weight. At the other end, loadings higher than 60 N resulted in a rapid deformation and excessive wear over a few minutes, in such a way that hardly any information (the kinetics of weight loss, for example) could be drawn from the test. The tests were continued up to 1000 m. Wear tests were interrupted after every 250 m to measure weight loss. The velocity of the moving pin on the disc was chosen to be a constant amount of 0.25 m/s. Wear debris, collected during wear tests, were also evaluated using SEM. SEM also investigated the surface topologies after wear tests.

## 3. Case Background

**Figure 1:**
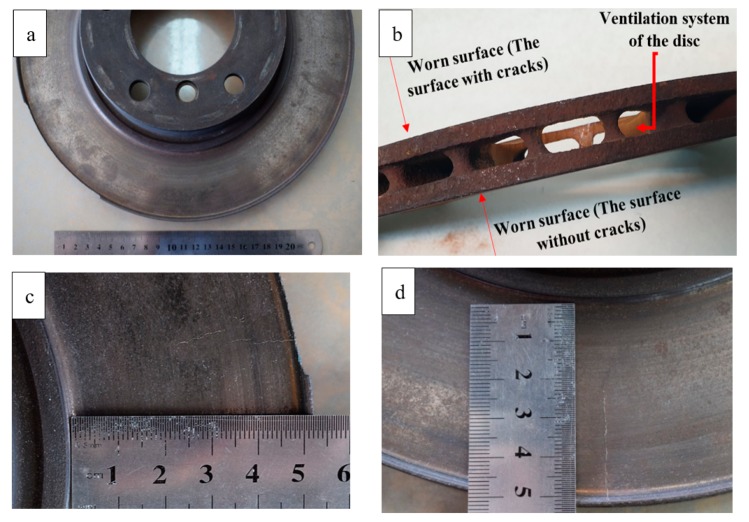
(**a**,**b**) Optical images of the disc and (**c**,**d**) images of the cracks at the surface of the brake disc.

The failure, in this case, was related to the gray cast iron (Fe-3.3% C, 2.1%Si, 2.2% Mn, 0.2%P and 0.1%S, given by the supplier) disc brake in an automobile (see Figure 1a,b). The failed disc had been in service for approximately 10 years. The mileage when the failure was reported was 100,000 km (10 years of service). The disc had been in operation in temperature variations between −10 °C (in winter) to 35 °C in summer in a rather dry atmosphere. The disc was a ventilated one and it was cast with two sides being in contact with the break-pad simultaneously. However, micro-cracks were formed only on one side. The observed failure was in the form of radial cracks, which appeared to be initiated at the outer edge of the brake disc. There were two rather large cracks, with one being approximately 30 mm and the other one being around 40 mm (see Figure 1c,d). The former crack was in fact two separated cracks, propagated in one direction, while the latter one was a straight continuous crack. There was no indication of the crack branching on the surface.

## 4. Results and Discussion

**Figure 2:**
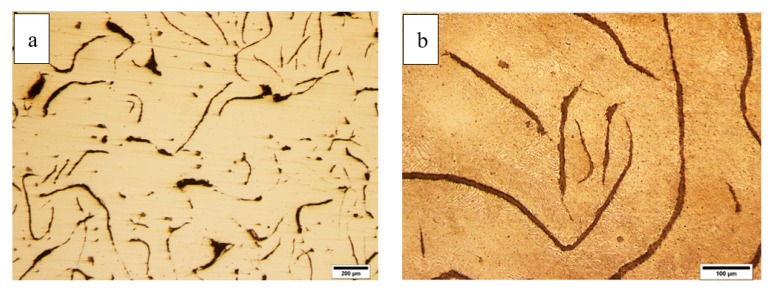
Optical microscope images of the microstructure in (**a**) as-polished and (**b**) etched conditions.

**Figure 3:**
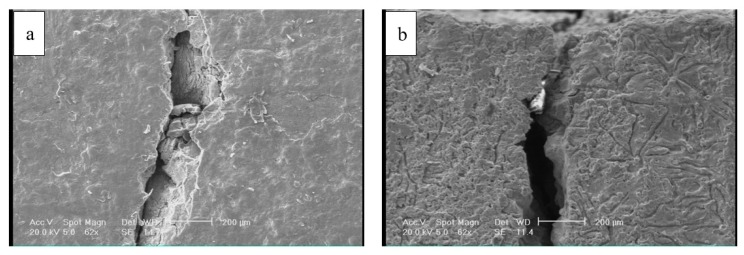
Scanning electron microscope (SEM) images of the crack, in (**a**) top view and (**b**) cross-section.

**Figure 4:**
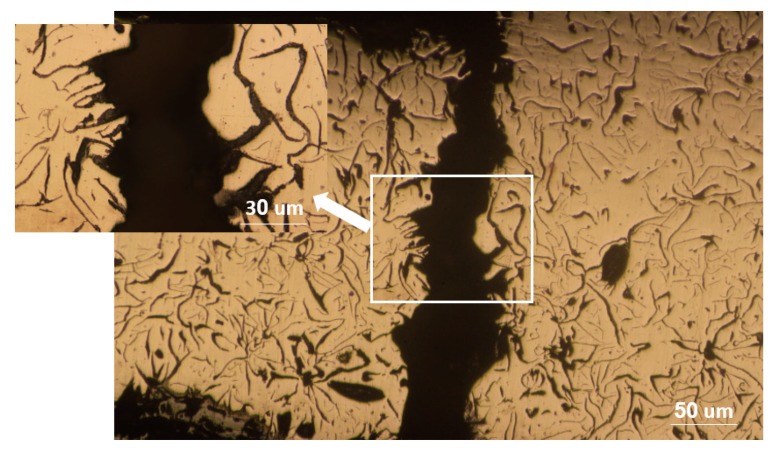
Optical microscope image of the cross-section of the crack.

**Figure 5:**
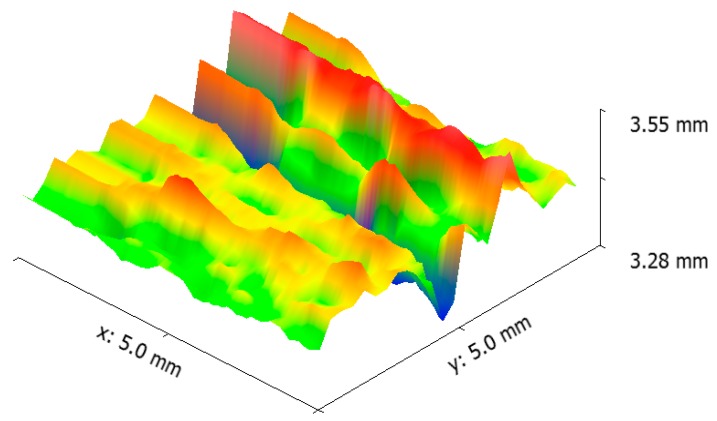
Laser profilometry of the disc brake surface in the vicinity of the crack.

Figure 2 shows optical microscope images of the disc before and after etching. As shown, graphite flakes were distributed in a pearlite matrix. Graphite flakes are excellent thermal conductors, making gray irons good thermal conductors as well. This also has to do with the fact that graphite flakes form an interconnected network, forming an easy path for heat transfer. Moreover, graphite flakes are known to absorb sounds created by the brake pad-brake disc contacts [20]. Figure 3 shows the SEM images of cracks on the surface of the disc from the top view and from the cross-section. The former image shows that crack openings on the surface in some areas were as large as 200 µm. It appeared that these large openings on the surface were formed due to the spallation of rather large chips from the surface. The remainder of these chip-like features were still visible inside the crack. Further away at the right side of the crack, there was a spot (roughly 200 µm × 100 µm), which was seemingly the area from which a surface chip spallation had taken place. Figure 3b and Figure 4 are images of the cross-section of the crack. There was no branching or any major deviation from the straight line. Figure 5 shows the laser profilometry of the surface topography in the vicinity of the crack. There were deep ups and downs parallel to each other [21]. Sharp edges on the surface were indications of surface cutting and abrasive wear on the surface.

**Figure 6:**
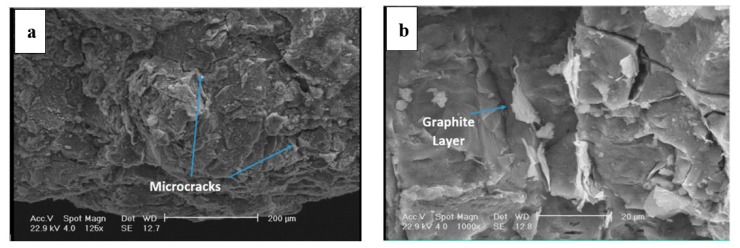
(**a**,**b**) SEM images of the fracture surface in the brake disc.

**Figure 7:**
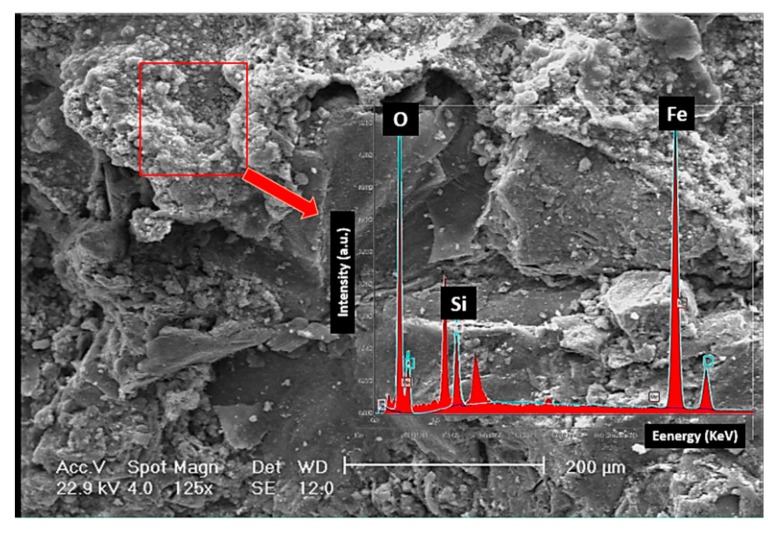
SEM/EDS analyses of the oxide particles.

Figure 6 shows the fracture surface of the disc. Fracture is overall brittle with a layered structure (see Figure 6a). The fracture surface was covered with a network of fine microcracks. The interface of matrix-graphite, in which matrix is referred to pearlite, is a suitable place for crack initiation, given that the adhesion of graphite to the iron-based structure is rather weak. An example is depicted in Figure 6b. It appears that the fracture had mostly initiated from the graphite/matrix interface. Figure 7 shows that the fracture surface in some areas is covered with fine agglomerated particles. EDS results show that these particles were mostly Fe- and Si-containing oxide particles. These particles were formed during the wear of the surface and detachment of the particles. This is while the high temperature of the disc during the contact of the pad and the disc resulted in the diffusion of the oxygen atoms into the lattice and the oxidation of the particles. Fine oxide particles have relatively higher hardness compared to the matrix. These particles can cause severe abrasion in case they are entrapped between two moving components, which in this case for the brake pad and the disc. The formation of fine oxide particles between the brake pad and the disc had to do with localized temperature increase due to the excessive friction at local contact points between the pad and the disc.

**Figure 8:**
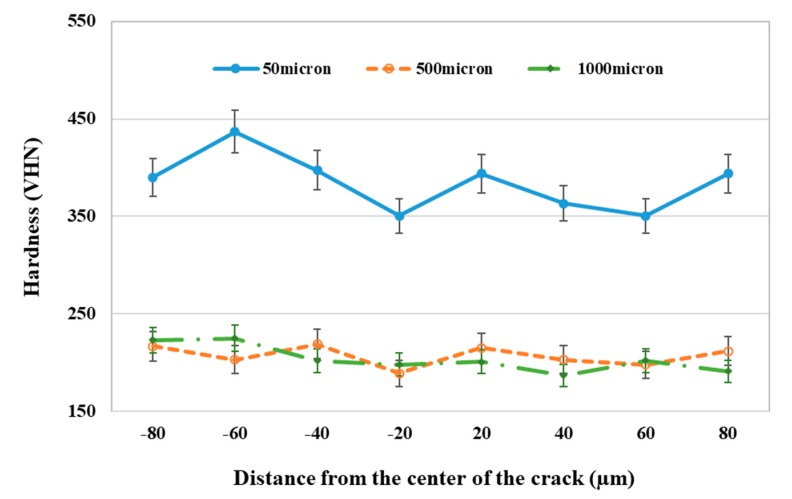
Hardness profilometry across the crack at different depths from the surface.

Results of microhardness profilometry across the crack at different depths from the surface are shown in Figure 8. Disc brakes experience large surface compression stresses due to exerted loads from the brake pad. This results in plastic deformation and work hardening at layers close to the surface. In this case, this resulted in an increase in the hardness from about 200 VHN at deep layers inside the brake disc to 400 VHN in layers close to the surface. This increase in the hardness confirmed the argument that the surface was heavily deformed and work-hardened during service. Prasad [4] also reported the occurrence of work hardening in gray cast iron discs during a wear test. The depth of the work-hardened layer was dependent on the loading conditions, the sliding distance (service time), and the temperature increase at local contact points.

**Figure 9:**
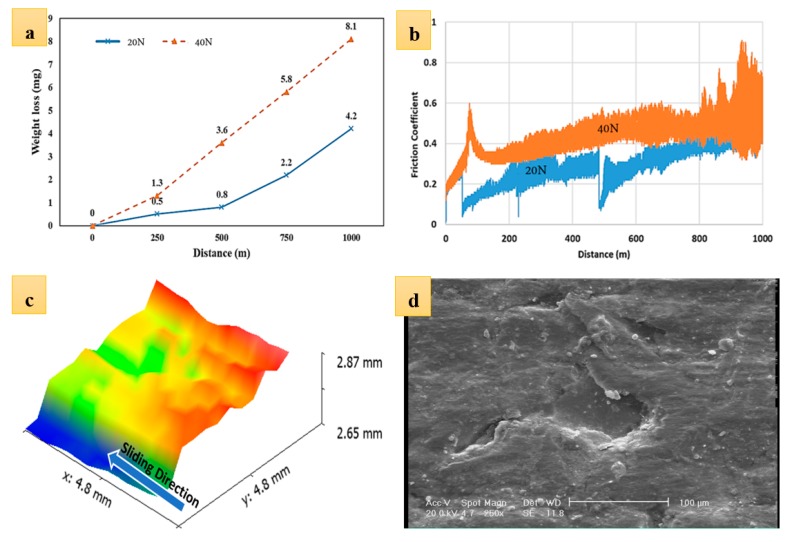
Results of the wear test: (**a**) weight loss with sliding distance, (**b**) variation of friction coefficient with sliding distance, (**c**) laser profilometry of the sample after 1000 m wear test under 40 N load, and (**d**) SEM image of the same sample.

To further investigate wear mechanisms in gray cast iron, pin-on-disc wear tests were performed on discs of 50 mm diameter. The tests were conducted for 1000 m under 20 and 40 N loads. Figure 9 depicts the results of the wear tests. Figure 9a compares the weight loss during wear tests under 20 and 40 N loads. The higher the load, the higher the weight loss. In the case of a 40 N load, the weight loss linearly increased with the sliding distance. However, in the case of a 20 N load, it appeared that the weight loss had different kinetics during the test, in such a way that the kinetics of weight loss was much slower in the first 500 m of sliding. The kinetics of weight loss significantly increased from this point on. This could be due to the activation of a new wear mechanism. Given that oxide particles have a significant contribution to the wear [22], one can postulate that up to 500 m, not enough oxide particles were formed in between the pin and disc. This changed after 500 m of sliding. This argument follows the variation of the friction coefficient with sliding distance (see Figure 9b). We see that where the load was 20 N, the coefficient of friction increased with different kinetics after 500 m. Concerning the coefficient of friction, one can see that the coefficient of friction with a load of 40 N was comparatively larger than that with a load of 20 N. This had to do with the correlation of the applied load and the coefficient of friction. As the applied load increased during the wear test, the contact points between the asperities increased, which resulted in a higher coefficient of friction. The observed increase in the coefficient of friction with sliding distance in both cases could be attributed to the formation and fragmentation of oxide particles on the surface. More importantly, as the sliding distance increased, the chance of detachment and fragmentation of the graphite flakes increased. Given that graphite flakes have a self-lubricating effect, this detachment and fragmentation of graphite flakes can increase the coefficient of friction. Graphite detachment can also increase the surface roughness and this will have a negative implication on the coefficient of friction. Figure 9c,d show the laser profilometry of the surface topography and SEM image of the worn surface after 1000 m sliding under load 40 N. There were different features on the surface, with each being related to different wear mechanisms. First of all, there were some spots on the surface wherein the surface had clearly undergone severe plastic deformation. In some areas, this resulted in spallation on the surface (see Figure 9d). Other than that, there were some plowings on the surface related to the abrasion due to the relative movement of oxide particles on the surface. Moreover, some surface cutting was identified on the surface. Cutting was identified when there was material separating with negligible plastic deformation at the sides of the grooves.

**Figure 10:**
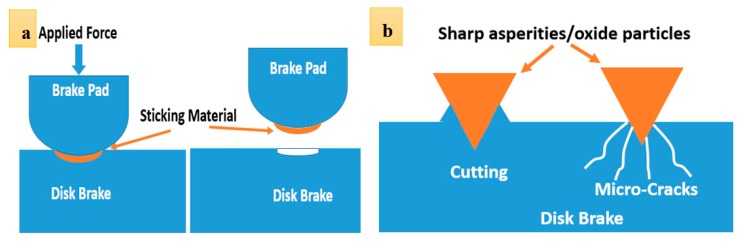
Schematics of (**a**) adhesive wear and (**b**) abrasive wear mechanisms.

**Figure 11:**
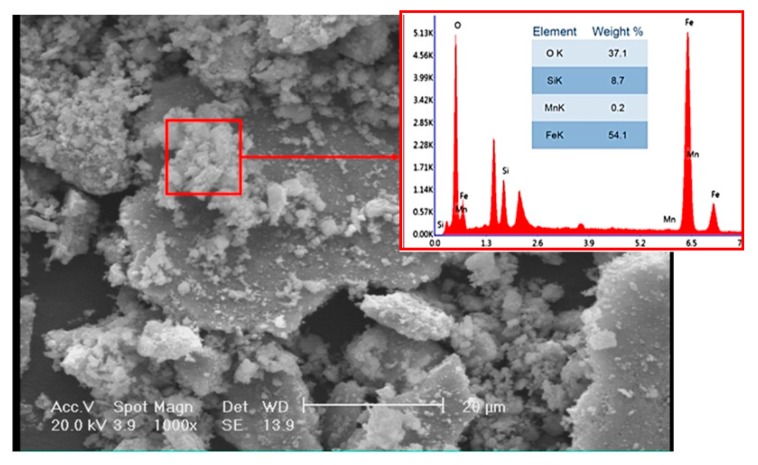
SEM image/EDS analysis of wear debris (x-axis: KeV, y-axis: a.u.).

While plastic deformation and spallation are indications of an adhesive wear mechanism, plowing and cutting can be linked to the abrasive wear, inferring that both adhesive and abrasive wear mechanisms contribute to weight loss during wear test. The former wear mechanism has a relatively higher kinetics of weight loss. In severe conditions, contact points in-between sliding bodies can be severely damaged by adhesive wear. In case the surface has some minimum roughness and the applied load is high enough, metal-metal sticking on contact spots becomes inevitable. In case the so-called sticking layer is weakly bonded to the disc, it will eventually be detached from the surface. This is schematically shown in Figure 10a. In comparison to the adhesive wear, abrasive wear takes place when a hard particle/asperity is sliding over a surface. Hard particles/asperities can cause abrasion without having to induce any major plastic deformation on the surface. In case particles/asperities are sharp enough, they create surface cutting. However, their attributions are not limited to cutting, as they could also cause surface micro-fractures and grain pull-outs (see Figure 10b) [23]. Cutting and micro-cracks are essentially more dominant features on the fracture surface. Surface cutting results in the detachment of wear debris. Where the wear debris is hard and fragile, they will be fragmented and entrapped in between sliding bodies and inside surface grooves, and, in turn, contribute to the abrasion of the surface. The attribution of wear debris to the wear rate depends on the relative hardness of wear particles to that of the worn surface. Fast abrasion is expected to take place when the hardness of the matrix is less than 80% of the hardness of particles [23]. If the load at the contact points is high enough, the temperature at the contact points increases, and therefore, oxidation of the wear debris is also likely to take place. This appears to be the case in this failure (see Figure 7). Oxide particles are clearly hard and are expected to cause further abrasion. Both cutting and micro-cracks are less probable when the worn surface is ductile in nature. Results show that in this case, both adhesive wear and abrasion control the wear of the disc brake during service. Figure 11 shows the SEM images of debris after wear tests. Debris can primarily be categorized into large plate-like chips, with their sizes being in the range of a few ten micrometers, and fine agglomerated particles. EDS analysis shows that these particles are oxide particles, thereby supporting the aforementioned argument concerning the formation of oxide particles due to temperature increases in-between the pin and the disc.

## 5. Conclusions

This paper investigated the root cause of crack formation in an automotive disc brake. Furthermore, laboratory pin-on-disc experiments were conducted to evaluate the wear mechanism of gray cast iron. The following conclusions can be drawn:

- The observed cracks were rather straight and appeared to be initiated from the outer surface of the disc and grew inwards towards the center of the disc. Cracks are formed due to the wear and severe loading conditions and are propagated perpendicular to the sliding direction.
- Inside cracks in some areas were filled with chip-like and fine spherical particles. These external particles inside cracks are characterized to be oxide particles in nature.
- Oxide particles were formed during the wear test as a result of temperature increases at brake pad-disc contacts. These oxide particles have significant attributions to abrasion and weight loss.
- Graphite-matrix interface detachment was observed on the fracture surface of the disc. Graphite detachment creates surface roughness and surface irregularities, which in turn increase the coefficient of friction.
- Both abrasive and adhesive wear mechanisms were identified in pin-on-disc samples.

## References

[1] Cueva G., Sinatora A., Guesser W., Tschiptschin A.J.W. (2003). Wear resistance of cast irons used in brake disc rotors. Wear.

[2] Rivera G.L., Boeri R.E., Sikora J.A. (2004). Solidification of gray cast iron. Scr. Mater..

[3] Zhang Y., Chen Y., He R., Shen B. (1993). Investigation of tribological properties of brake shoe materials-phosphorous cast irons with different graphite morphologies. Wear.

[4] Cho M.H., Kim S.J., Basch R.H., Fash J.W., Jang H. (2003). Tribological study of gray cast iron with automotive brake linings: The effect of rotor microstructure. Tribol. Int..

[5] Wang W., Jing T., Gao Y., Qiao G., Zhao X. (2007). Properties of a gray cast iron with oriented graphite flakes. Mater. Process. Technol..

[6] ELSawy E.E.T., EL-Hebeary M.R., El Mahallawi I.S.E. (2017). Effect of manganese, silicon and chromium additions on microstructure and wear characteristics of gray cast iron for sugar industries applications. Wear.

[7] Jimbo Y., Mibe T., Mihe T., Akiyama K., Matsui H., Yoshida M., Ozawa A.J.S.T. (1990). Development of High Thermal Conductivity Cast Iron for Brake Disc Rotors.

[8] Keller J., Fridrici V., Kapsa P., Vidaller S., Huard J.J.W. (2017). Influence of chemical composition and microstructure of gray cast iron on wear of heavy duty diesel engines cylinder liners. Wear.

[9] Prasad B.K. (2011). Sliding wear response of a gray cast iron: Effects of some experimental parameters. Tribol. Int..

[10] Prasad B.K. (2008). Sliding wear characteristics of a gray cast iron as influenced by the sliding speed, load and environment. Tribol. Mater. Surf. Interfaces.

[11] Gowda D., Charan Kumar D., Sandeep G.M., Parthasarathy A., Chandrashekar S. (2018). Tribological Characterization of Centrifugally Cast Graphite Cast Iron under Dry and Wet conditions. Mater. Today Proc..

[12] Jang H., Ko K., Kim S., Basch R., Fash J.J.W. (2004). The effect of metal fibers on the friction performance of automotive brake friction materials. Wear.

[13] White C.V. (1990). Gray Iron. Metals Handbook: Properties and Selection: Irons, Steels and High Performance Materials.

[14] Eyre T.S. (1992). Friction and wear of cast irons. Metals Handbook: Friction, Lubrication and Wear Technology.

[15] Makar J., Desnoyers R., McDonald S.E. (2001). Failure Modes and Mechanisms in Gray Cast Iron Pipe, Institute for Research in Construction.

[16] Bagnoli F., Dolce F., Bernabei M.J.E.F.A. (2009). Thermal fatigue cracks of fire fighting vehicles gray iron brake discs. Eng. Fail. Anal..

[17] Yang Z.Y., Han J.M., Li W.J., Li Z.Q., Pan L.K., Shi X.L. (2013). Analyzing the mechanism of fatigue crack initiation and propagation in CRH EMU brake discs. Eng. Fail. Anal..

[18] McPhee A.D., Johnson D.A. (2008). Experimental heat transfer and flow analysis of a vented brake rotor. Int. J. Therm. Sci..

[19] Gao C.H., Huang J.M., Lin X.Z., Tang X.S. (2007). Stress analysis of thermal fatigue fracture of brake discs based on thermomechanical coupling. J. Tribol..

[20] Collini L., Nicoletto G., Konečná R.J.M.S. (2008). Microstructure and mechanical properties of pearlitic gray cast iron. Mater Sci. Eng. A.

[21] Bathe R., Krishna V.S., Nikumb S., Padmanabham G.J.A.P.A. (2014). Laser surface texturing of gray cast iron for improving tribological behavior. Mater Sci. Process..

[22] Bahrami A., Mousavi Anijdan S.H., Golozar M.A., Shamanian M., Varahram N. (2005). Effects of conventional heat treatment on wear resistance of AISI H13 tool steel. Wear.

[23] Findik F. (2014). Latest progress on tribological properties of industrial materials. Mater. Des..

